# Supplementary material for: Intravenous versus inhalational maintenance of anesthesia for quality of recovery in adult patients undergoing non-cardiac surgery: A systematic review with meta-analysis and trial sequential analysis
Source: PLoS One. 2021 Jul 16;16(7):e0254271. doi: 10.1371/journal.pone.0254271 (PMC8284831; doi:10.1371/journal.pone.0254271)
Supplement: S1 File — (DOCX) [file pone.0254271.s002.docx]

**PubMed**

**((((quality of recovery[Title/Abstract]) OR (QoR[Title/Abstract])) OR (QoR-40[Title/Abstract])) AND ((((((Anesthetics, Inhalation[MeSH Terms]) OR (Anesthesia, Inhalation[MeSH Terms])) OR (Sevoflurane[MeSH Terms])) OR (Desflurane[MeSH Terms])) OR (Isoflurane[MeSH Terms])) OR (Balanced Anesthesia[MeSH Terms]))) AND (((((Anesthesia, Intravenous[MeSH Terms]) OR (Anesthesias, Intravenous[Title/Abstract])) OR (Intravenous Anesthesia[Title/Abstract])) OR (Intravenous Anesthesias[Title/Abstract])) OR (total intravenous anesthesia[Title/Abstract]))**

**EMBASE**

#1. 'intravenous anesthesia'/exp OR 'intravenous

anesthesia*' OR 'tiva' OR 'total intravenous

anesthesia' OR 'anesthesia, intravenous'

#2. 'balanced anesthesia'/exp OR 'inhalation

anesthetic agent'/exp OR 'inhalation

anesthesia'/exp OR 'sevoflurane'/exp OR

'desflurane'/exp OR 'isoflurane'/exp

#3. 'quality of recovery 40 questionnaire'/exp OR

'quality of recovery 40 questionnaire' OR

'quality of recovery' OR 'qor' OR 'qor-40'

#4. #1 AND #2 AND #3

**Cochrane**

#1 MeSH descriptor: [Anesthesia, Intravenous] explode all trees

#2 MeSH descriptor: [Anesthetics, Intravenous] explode all trees

#3 (Anesthesias, Intravenous):ti,ab,kw OR (Intravenous Anesthesia):ti,ab,kw OR (total intravenous anesthesia):ti,ab,kw OR (TIVA):ti,ab,kw OR (Intravenous Anesthesias):ti,ab,kw

#4 #1 OR #2 OR #3

#5 MeSH descriptor: [Balanced Anesthesia] explode all trees

#6 MeSH descriptor: [Anesthetics, Inhalation] explode all trees

#7 MeSH descriptor: [Anesthesia, Inhalation] explode all trees

#8 MeSH descriptor: [Sevoflurane] explode all trees

#9 MeSH descriptor: [Desflurane] explode all trees

#10 MeSH descriptor: [Isoflurane] explode all trees

#11 #5 OR #6 OR #7 OR #8 OR #9 OR #10

#12 (quality of recovery):ti,ab,kw OR (QoR-40):ti,ab,kw OR (QoR):ti,ab,kw

#13 #4 AND #11 AND #12 in Trials
