# Supplementary material for: Intravenous versus inhalational maintenance of anesthesia for quality of recovery in adult patients undergoing non-cardiac surgery: A systematic review with meta-analysis and trial sequential analysis
Source: PLoS One. 2021 Jul 16;16(7):e0254271. doi: 10.1371/journal.pone.0254271 (PMC8284831; doi:10.1371/journal.pone.0254271)
Supplement: S2 File — (DOCX) [file pone.0254271.s003.docx]

| **Intravenous versus inhalational maintenance of anesthesia for quality of recovery in adult patients** | | | | | | |
| --- | --- | --- | --- | --- | --- | --- |
| **Patient or population:** adult participants undergoing non-cardiac surgery under general anesthesia **Settings:** postoperative care in hospital, China (three trials), USA (two trials), Korea (two trials), Brazil (two trials) **Intervention:** TIVA **Comparison:** Inhalational maintenance | | | | | | |
| **Outcomes** | **Illustrative comparative risks* (95% CI)** | | **Relative effect (95% CI)** | **No of Participants (studies)** | **Quality of the evidence (GRADE)** | **Comments** |
|  | Assumed risk | Corresponding risk |  |  |  |  |
|  | **Inhalational maintenance** | **TIVA** |  |  |  |  |
| **Total QoR-40 on the day of surgery** |  | The mean total qor-40 on the day of surgery in the intervention groups was **5.91 higher** (2.14 to 9.68 higher) |  | 193 (3 studies) | ⊕⊕⊝⊝ **low**^1^ |  |
| **Total QoR-40 on POD1** |  | The mean total qor-40 on pod1 in the intervention groups was **1.65 higher** (0.46 lower to 3.75 higher) |  | 839 (8 studies) | ⊕⊕⊝⊝ **low**^2^ |  |
| **Physical comfort 6h after surgery** |  | The mean physical comfort 6h after surgery in the intervention groups was **1.49 higher** (0.14 to 2.84 higher) |  | 163 (2 studies) | ⊕⊕⊝⊝ **low**^1^ |  |
| **Physical comfort on POD1** |  | The mean physical comfort on pod1 in the intervention groups was **0.62 higher** (0.93 lower to 2.16 higher) |  | 454 (5 studies) | ⊕⊝⊝⊝ **very low**^3,4,5^ |  |
| **Emotional status 6h after surgery** |  | The mean emotional status 6h after surgery in the intervention groups was **1.82 higher** (0.75 to 2.89 higher) |  | 163 (2 studies) | ⊕⊕⊝⊝ **low**^1^ |  |
| **Emotional status on POD1** |  | The mean emotional status on pod1 in the intervention groups was **0.31 higher** (0.19 lower to 0.82 higher) |  | 454 (5 studies) | ⊕⊕⊝⊝ **low**^4,5^ |  |
| **Psychological support 6h after surgery** |  | The mean psychological support 6h after surgery in the intervention groups was **1.4 higher** (0.5 to 2.29 higher) |  | 163 (2 studies) | ⊕⊕⊝⊝ **low**^1^ |  |
| **Psychological support on POD1** |  | The mean psychological support on pod1 in the intervention groups was **0.39 higher** (0.48 lower to 1.26 higher) |  | 454 (5 studies) | ⊕⊝⊝⊝ **very low**^4,6^ |  |
| **Physical independence 6h after surgery** |  | The mean physical independence 6h after surgery in the intervention groups was **1.49 higher** (0.36 to 2.62 higher) |  | 163 (2 studies) | ⊕⊕⊝⊝ **low**^1^ |  |
| **Physical independence on POD1** |  | The mean physical independence on pod1 in the intervention groups was **0.47 higher** (0.45 lower to 1.38 higher) |  | 454 (5 studies) | ⊕⊝⊝⊝ **very low**^4,5,7^ |  |
| **Pain 6h after surgery** |  | The mean pain 6h after surgery in the intervention groups was **0.55 higher** (0.02 lower to 1.13 higher) |  | 163 (2 studies) | ⊕⊕⊝⊝ **low**^1^ |  |
| **Pain on POD1** |  | The mean pain on pod1 in the intervention groups was **0.31 lower** (0.74 lower to 0.13 higher) |  | 454 (5 studies) | ⊕⊕⊝⊝ **low**^4,5^ |  |
| *The basis for the **assumed risk** (e.g. the median control group risk across studies) is provided in footnotes. The **corresponding risk** (and its 95% confidence interval) is based on the assumed risk in the comparison group and the **relative effect** of the intervention (and its 95% CI).  **CI:** Confidence interval; | | | | | | |
| GRADE Working Group grades of evidence **High quality:** Further research is very unlikely to change our confidence in the estimate of effect.  **Moderate quality:** Further research is likely to have an important impact on our confidence in the estimate of effect and may change the estimate. **Low quality:** Further research is very likely to have an important impact on our confidence in the estimate of effect and is likely to change the estimate. **Very low quality:** We are very uncertain about the estimate. | | | | | | |
| ^1^ Few studies with few participants; we downgraded by one level for imprecision and one level for publication bias.  ^2^ Overall, 10 studies (1100 participants) reported data for this outcome. 1 study reported data with QoR-15 questionnaire that could not be combined with other studies (we noted no difference between groups). 1 study with high risk of bias and significantly increasing heterogeneity was also excluded. According to the trial sequential analysis, the sample size did not reach the required information size. We downgraded by one level for imprecision and one level for publication bias.  ^3^ We downgraded by one level for inconsistency (I2 = 63%).  ^4^ We downgraded by one level for imprecision (not reaching the required information size). ^5^ We downgraded by one level for publication bias (asymmetrical funnel plot).  ^6^ We downgraded by two levels for inconsistency; we noted substantial statistical heterogeneity (I2 = 81%).  ^7^ We downgraded by two levels for inconsistency; we noted substantial statistical heterogeneity (I2 = 77%). | | | | | | |
