# Supplementary material for: Intravenous versus inhalational maintenance of anesthesia for quality of recovery in adult patients undergoing non-cardiac surgery: A systematic review with meta-analysis and trial sequential analysis
Source: PLoS One. 2021 Jul 16;16(7):e0254271. doi: 10.1371/journal.pone.0254271 (PMC8284831; doi:10.1371/journal.pone.0254271)
Supplement: S3 File — (DOCX) [file pone.0254271.s004.docx]

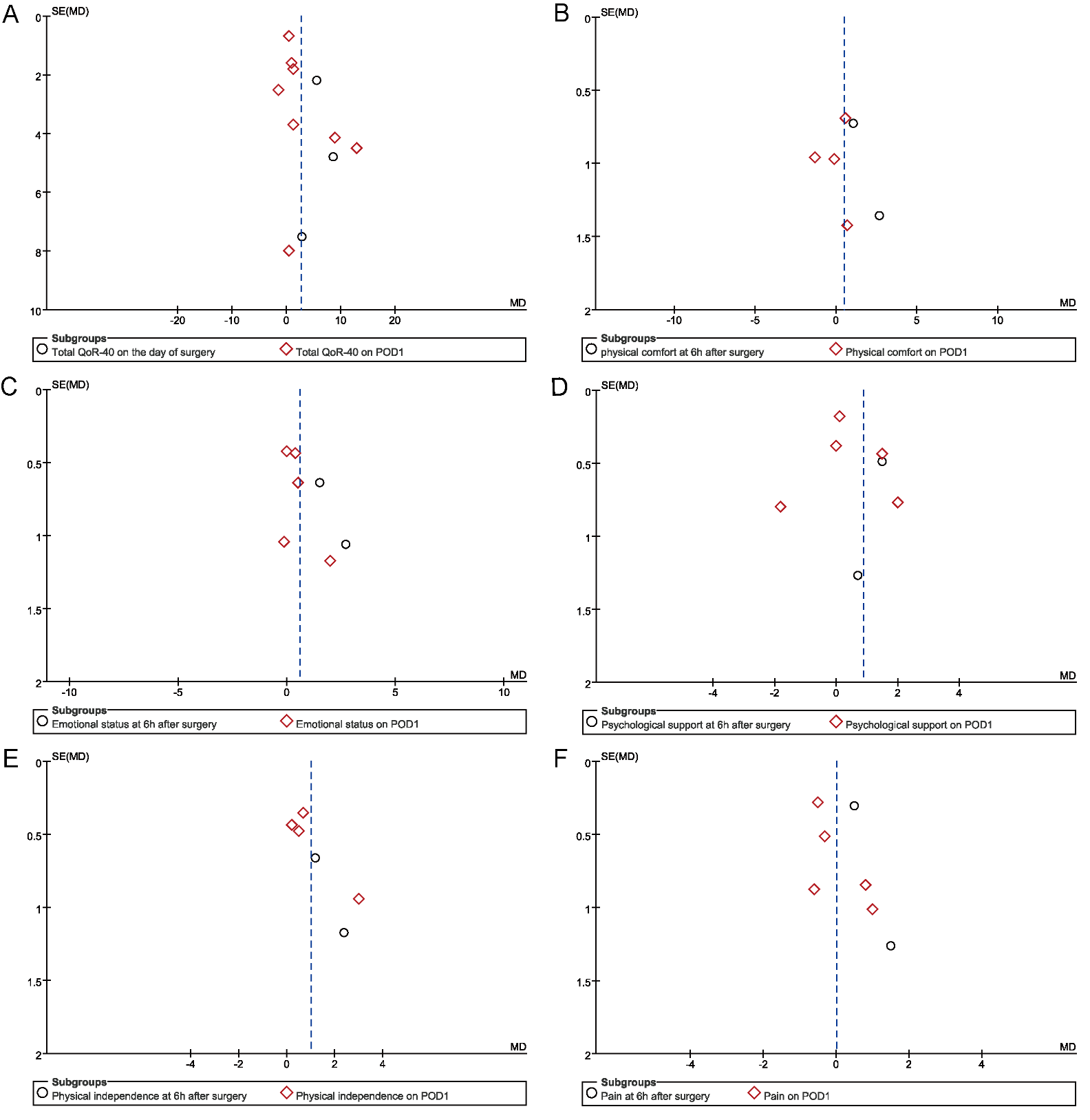


Funnel plot for total QoR-40 (A), physical comfort (B), emotional status (C), psychological support (D), physical independence (E) and pain (F) with TIVA versus inhalational maintenance. SE, standard error; MD, mean difference; POD1, postoperative day 1.
