# Supplementary material for: Intravenous versus inhalational maintenance of anesthesia for quality of recovery in adult patients undergoing non-cardiac surgery: A systematic review with meta-analysis and trial sequential analysis
Source: PLoS One. 2021 Jul 16;16(7):e0254271. doi: 10.1371/journal.pone.0254271 (PMC8284831; doi:10.1371/journal.pone.0254271)
Supplement: Attachment — Submitted filename: Response to Reviewers.docx [file pone.0254271.s005.docx]

Reviewer #1: The authors conducted a meta-analysis with trial sequential analysis to determine if total intravenous anesthesia was better than inhalation anesthetics in global recovery of patients. The clinical trials included in this meta-analysis used Quality of Recovery-40 score (QoR 40). The primary studies mostly involved mostly females, patients undergoing outpatient or ambulatory surgeries, intermediate to low risk surgeries. This is an important question for anesthesiologist, which to date has generated inconclusive results. This study attempts to combine results from nine studies and in essence reaches the same conclusion that the results are inconclusive.

General Comments

1. Though the total number of studies is 9 and n=922 patients, in reality there are two types of studies. One group of studies reporting results by six hours which has n of 193 and a second group reporting results by postoperative day one with an n= 839. The smaller group shows some benefit of total intravenous anesthesia, but the larger group does not show a difference in global recovery as reported by by QoR 40 score. Both groups do not achieve RIS threshold, so based on trial sequential analysis one can consider that the meta-analysis is under-powered to date.
From reported evidence, our meta-analysis and trial sequential analysis, it is true that we couldn’t draw a firm conclusion, but it is real and it can provide reference for future research.

2. All studies did not report all domains and sub-analysis of domains contain even less number of patients, which highlight another limitation of the study.
We couldn’t agree more. These limitations were discussed in our manuscript.

3. Trial Sequential analysis have their own limitations and it may be helpful for readers if the authors highlight those limitations (see Anaesthesia 2020, 75, 15-20) and explain how they have addressed some of the methodological limitation of the study.
It is true that trial sequential analysis (TSA) have their own limitations. The TSA aimed to make the conclusion more reliable. And we complied with its rules. Thus, we think there may be no need to discuss the limitations of TSA in this meta-analysis.

4. This study provides a framework for future studies and I suspect, as with many meta-analyses, this is probably one of the initial ones in preparation for other analysis in future.
There seems no requirement for revision in this comment.

5. In my opinion considering that the studies showing benefit of total intravenous anesthesia are limited to six hours and have less than 200 patients, it would be advisable to tone down the benefit of total intravenous anesthesia versus inhalational anesthetic and highlight more than methodology, it's limitations and inconclusive nature of this question so far.
Our meta-analysis and TSA results clearly showed that current evidence couldn’t draw a firm conclusion. The current evidence does not support that total intravenous anesthesia is more advantageous.

6. Another point to highlight is that the difference in absolute score is very small and its clinical significance is not clear.
In our meta-analysis, trial sequential analysis showed that the difference in absolute score was not established, its clinical significance is certainly not clear. Thus, more evidence is needed. That was our conclusion.

Specific comments

P4 L71: What is meant by ‘quality of healthcare resources”?
We revised it in the manuscript.
P4 L81: …’discomfort’ sounds very informal. May be authors may consider ‘ …. various factors impact quality of recovery of patients after surgery. ‘
We revised it in the manuscript.
Figure 4: Post-operative Day 1 Physical independence
The total n should be 229 and 225. Please amend.

The study (De Carli 2020) was not estimable. Thus, the total number is right.

Reviewer #2: Title:

Intravenous versus inhalational maintenance of anesthesia for quality of recovery in adult patients
Suggested: Intravenous versus inhalational maintenance of anesthesia for quality of recovery in adult patients
undergoing non-cardiac surgery.

We revised it in the manuscript.

Abstract:
Background: Second line: It is still controversial as to which technique
Suggested: It is still controversial which technique

We revised it in the manuscript.
Third line: This meta-analysis aimed to compare impact
Suggested: This meta-analysis aimed at comparing the impact

We revised it in the manuscript.
Methods:
Line 8/9: estimated required information size for total QoR-40 scores were not surpassed by
recovered evidence in our meta-analysis
Suggested: estimated required information size for total QoR-40 scores was not surpassed by
recovered evidence in our meta-analysis.

We revised it in the manuscript.
Page 5
All relevant data are within the manuscript and its Supporting Information files.
Suggested: All relevant data are within the manuscript and its supporting information files
We revised it in the manuscript.


Body of manuscript:
Line 25: It is still controversial as to which technique
Suggested: It is still controversial which technique

We revised it in the manuscript.
Line 26: This meta-analysis aimed to compare impact
Suggested: this meta-analysis aimed at comparing the impact

We revised it in the manuscript.
Line 69: The introduction mentioned (ambulatory anaesthesia) while the title is not about ambulatory
anaesthesia??!
Line 80: Please, omit reference number 7 and omit it from the references list as it is related to paediatric
anaesthesia and the scope of the manuscript is adult anaesthesia.

We revised it in the manuscript.
Line 127: Kindly, (Supplementary) to be written as (supplementary)

We revised it in the manuscript.
Line 132/133:
Here the 9 included studies used the intravenous induction route so the manuscript compares TIVA with
inhalational maintenance, so inhalational induction should be omitted.

We revised it in the manuscript.
Line 258: Kindly, change (Supplementary) to be (supplementary).
We revised it in the manuscript.
Discussion:


Lines 298/299:
more evidence is needed for a firm conclusion. Then we need to consider the clinical significance.
Suggested:
more evidence is needed for a firm conclusion, then we need to consider the clinical significance.

We revised it in the manuscript.

Line 309:
It might due to the mean difference (95% CI) too close to the invalid line
Suggested:
It might be due to the mean difference (95% CI) was too close to the invalid line
We revised it in the manuscript.
Line 333:
meta-analysis to address patient perception of postoperative recovery quality between the two anaesthetic
techniques.
Suggested:
meta-analysis to address patient perception of postoperative quality of recovery of the two anaesthetic
techniques.
We revised it in the manuscript.
Line 344:
participants of most studies were female
Suggested:
participants of most studies were females
We revised it in the manuscript.
Line 354:
And including these studies in future review updates would increase certainity of the effect.
Suggested:
And including these studies in future review updates will increase certainity of the effect.
We revised it in the manuscript.


References:

Reference number 19 link is invalid

We coped the link to the browser and opened it. It is right and valid.
