# Supplementary material for: Intravenous versus inhalational maintenance of anesthesia for quality of recovery in adult patients undergoing non-cardiac surgery: A systematic review with meta-analysis and trial sequential analysis
Source: PLoS One. 2021 Jul 16;16(7):e0254271. doi: 10.1371/journal.pone.0254271 (PMC8284831; doi:10.1371/journal.pone.0254271)
Supplement: Attachment — Submitted filename: Response to Reviewers2.docx [file pone.0254271.s006.docx]

Reviewer #2: Comments:

1- Line 69 and 70:
Requirement for effectiveness and efficiency of healthcare resources prompts anesthesiologists to consider techniques that provide fast and high quality of recovery.

Suggested (kindly, observe that there are 2 suggested changes in the statement)
The requirement for effectiveness and efficiency of healthcare resources prompts anesthesiologists to consider techniques that provide a fast and high quality of recovery.
We revised it in the manuscript.

2- Page 4 line 80 and 81:
In recent years researchers have recognized the limitations of the fragmentary measures....
Suggested: In recent years, researchers have recognized the limitations of the fragmentary measures...
We revised it in the manuscript.

3- Please, review the tables in page 12 and 13, the reference numbers are different from the numbers of the references written at the end of the manuscript.
We revised it in the manuscript.

4- Line 313/314: It might due to the mean difference (95% CI) was too close to the invalid line
suggested: It might be due to the mean difference (95% CI) was too close to the invalid line
We revised it in the manuscript.

5- Line 323: there is an extra space just before the word Herling, please omit this extra space.

We revised it in the manuscript.

Reviewer #3: Thank you for the opportunity to review the metanalysis entitled " Intravenous versus inhalational maintenance of anesthesia for quality of recovery in
adult patients undergoing non-cardiac surgery: a systematic review with meta-analysis and trial sequential analysis"

1) The premise of the analysis is interesting. However, there are concerning points that may/may not be fixable at this point. Important or possibly contentious question:
2) The included studies differ in the time of outcomes analysis - in the majority - smaller sized studies favored TIVA (are ambulatory/short outcome measure time) in addition represent inadequate work in this domain whereas other relatively larger studies showed “no effect”- plus studies are primarily female gender predominant together making it non-reproducible and more susceptible to selection bias. he problem with this analysis — in theory, you can select the trials you want to include and add them to come up with a number, but the question here is -would the average number apply to these diverse interventions which were compared with user-variable, by using different definitions of outcomes.
We agree with these limitations. We conducted the present meta-analysis also due to these controversies. And we just combined data with similar time point.

3) Introduction: Authors did not justify the rationale of the study. The logic "Wanders" and practically ends in the middle of a thought. Why do authors think that a metanalysis is necessary? Please explain the gaps of knowledge in introduction.
We revised it in the last paragraph in introduction.

4) Very likely that your analysis is not powered to answer this question. I am sure you will agree that the standards for metanalysis should be higher in such questions due to possible impact on the varying clinical practice and choice of anesthetic agents. The ‘required information size’ necessary to account for heterogeneity and multiple comparisons when the RCTs are added, the necessary TSA boundaries are not reached. Moreover, power should be defined apriori.
There seems no requirement for revision in this comment.

5) Please add power analysis and sample size calculation.
The estimated required information size (RIS) in TSA results was sample size calculation.

6) After reading the conclusion, some may overinterpret the results--therefore avoid making any strong statements and include—"future research is likely to have an important impact."

We revised it in the manuscript.

7) Authors stated the conclusions “strongly” when in fact nothing can be concluded so far for sure. They do have a signal but the results remain inconclusive; considering limitations as discussed above in point 1 and 2. At most the authors should state the “results are inconclusive due to limited power”

We revised it in the manuscript.

8) It would be helpful if you show the results of sensitivity analysis. Please clarify what are duplicate studies?
We showed the summarized results and alterations of sensitivity analysis. In the sensitivity analysis, we alternated meta-analytic effects model and excluded each study sequentially in score of each dimension. The results included too much single figures, so we did not upload these figures. If there is a need, we can submit the figures for supplementary files.

9) Please include the time period of your search, your last search date, and last metanalysis done.

It showed in the “Search Strategy and Study Selection” part.

10) Please name the random effect model used or cite the exact model.
We revised it in the manuscript.

11) Non-cardiac surgeries in title may be a bit misleading. Non-cardiac surgeries can vary a lot with all respect. Authors should consider mentioning something like “Same-day surgeries”
 We had considered the problem, but not all studies included in our meta-analysis were ‘same-day surgeries’. When we registered the meta-analysis, we did plan to perform a meta-analysis about non-cardiac surgeries.

12) Discussion: Similar to introduction. Too wordy and not focused to results. I would suggest reframing it according to the obtained results and just reporting the facts focused on the analysis. Plus, various confusing sentence wording and run-on sentences exist that should be restructured throughout. Awkward phrasing throughout the manuscript detracts from the overall quality of the manuscript. Limitation section should be a thoughtful list that caution your results.

We revised it in the manuscript.
