# Supplementary material for: Intravenous versus inhalational maintenance of anesthesia for quality of recovery in adult patients undergoing non-cardiac surgery: A systematic review with meta-analysis and trial sequential analysis
Source: PLoS One. 2021 Jul 16;16(7):e0254271. doi: 10.1371/journal.pone.0254271 (PMC8284831; doi:10.1371/journal.pone.0254271)
Supplement: Attachment — Submitted filename: response to reviewer3.docx [file pone.0254271.s007.docx]

Reviewer #2: This comment is repeated (written and sent to the author before), as there were 2 modifications included but the author did one only, so please, do the second modifications.

- Line 69 and 70:

Requirement for effectiveness and efficiency of healthcare resources prompts anesthesiologists to consider techniques that provide fast and high quality of recovery.

Suggested (kindly, observe that there are 2 suggested changes in the statement)

The requirement for effectiveness and efficiency of healthcare resources prompts anesthesiologists to consider techniques that provide a fast and high quality of recovery.

Originally in the last revision, I put the same comment, however you responded to one change only which is the addition of (The) to the start of the statement. There was another change which is the addition of (a) before (fast and high quality of recovery.).

We added (a) before (fast and high quality of recovery.)
